# Emergency cholecystectomy and hepatic arterial repair in a patient presenting with haemobilia and massive gastrointestinal haemorrhage due to a spontaneous cystic artery gallbladder fistula masquerading as a pseudoaneurysm

**DOI:** 10.1186/1471-230X-13-43

**Published:** 2013-03-03

**Authors:** Hazrah Priya, Gupta Anshul, Tiwari Alok, Kale Saurabh, Nath Ranjit, Lal Romesh, Sharma Deborshi

**Affiliations:** 1 Department of Surgery, Lady Hardinge Medical College, New Delhi, India; 2 Department of Surgery, Dr Ram Manohar Lohia Hospital, New Delhi, India; 3 Department of Cardiology, Dr Ram Manohar Lohia Hospital, New Delhi, India

**Keywords:** Cystic artery, Pseudoaneurysm, Haemobilia, Cholecystitis, Gastrointestinal, Haemorrhage

## Abstract

**Background:**

Haemobilia usually occurs secondary to accidental or iatrogenic hepatobiliary trauma. It can occasionally present with cataclysmal upper gastrointestinal haemorrhage posing as a life threatening emergency. Haemobilia can very rarely be a complication of acute cholecystitis. Here we report a case of haemobilia manifesting as massive gastrointestinal haemorrhage in a patient without any prior history of biliary surgery or intervention and present a brief review of literature.

**Case presentation:**

A 22 year old male admitted with history suggestive of acute cholecystitis subsequently developed waxing waning jaundice and recurrent episodes of upper gastrointestinal bleed. Endoscopy showed an ulcer in the first part of duodenum with a clot, no active bleed was visible. Angiography was suggestive of a ruptured pseudoaneurysm in the vicinity of the right hepatic artery probably originating from the cystic artery. Coil embolization was tried but the coil dislodged into the right branch of hepatic artery distal to the site of pseudoaneurysm. Review of angiographic video in light of operative findings demonstrated a fistulous communication between cystic artery and gallbladder as the cause, a simultaneous cholecystoduodenal fistula was also noted. Retrograde cholecystectomy, closure of cholecystoduodenal fistula and right hepatic arteriotomy with retrieval of the endo-coil and hepatic arterial repair was performed.

**Conclusion:**

Fistula between the cystic artery and gallbladder has been commonly reported to occur after laparoscopic cholecystectomy. Spontaneous fistulous communication, i.e. in the absence of any prior trauma or intervention, between cystic artery and gallbladder is rare with very few reports in literature. Aetiopathogenesis of the disease, in the context of current literature is reviewed. The diagnostic dilemma posed by the confounding finding of an ulcer in the duodenum, the iconic video angiographic depiction as also the therapeutic challenge of a failed embolization with consequent microcoil migration and primary hepatic arterial repair in the emergency situation is discussed.

## Background

Sandblom (1948) first coined the term haemobilia which literally implies blood in the biliary tract. The classical triad of right upper abdominal pain, jaundice and gastrointestinal bleeding is pathognomic of haemobilia. However haemobilia can present atypically primarily as gastrointestinal bleed or pancreatitis. Gastrointestinal haemorrhage though often mild, can at times be massive and present as an acute life threatening condition and is the most dreaded presentation of the disease.

Over the decades the aetiology, diagnosis and management of haemobilia has witnessed a tectonic transition. Trauma secondary to blunt or penetrating injuries, hepatobiliary surgery and or interventions has emerged as the most common cause of haemobilia (45%-85%) [1]. Some of the important non traumatic causes are: cholelithiasis, acalculous inflammatory diseases, vascular disorders, and neoplasms [1,2]. Vascular aneurysm especially of the cystic artery is an extremely rare cause of haemobilia. With the advent of laparoscopic cholecystectomy, post-surgical pseudoaneurysms of cystic artery have been increasingly reported [3,4]. However a spontaneous cystic artery pseudoaneurysm or fistula is still a rare cause of haemobilia. Spontaneous in the context is being referred to a non-traumatic aetiology i.e. in the absence of prior surgical or non-surgical trauma or interventions. Selective arterial angiography and embolization has emerged as an important tool to help localize the source of the bleed and aid in the management [2]. However the procedure can be misleading and may be occasionally wrought with complications. Here we describe a patient who had presented with massive upper gastrointestinal haemorrhage due to a spontaneous fistula between the cystic artery and gall bladder and discuss the confounding issues that were encountered in the diagnosis and management with particular reference to the use of angiography and embolization procedures.

## Case presentation

A 22 year old male presented to the emergency with history of severe colicky non-radiating pain in right upper abdomen of 7 days duration along with episodes of non-bilious vomiting. There was no history of fever, haematmesis, melana or any alteration of bowel or bladder habits. Past history and personal history were non-contributory. Mild icterus was noted and a tender gallbladder lump was palpable. His blood investigations at admission revealed a Hb of 11.9 gm/dl, TLC of 15500/mm [3], total bilirubin was 4.5 gm/dl, ALT 162 IU, AST 160 IU, Alk PO4 628 IU, an abdominal sonography showed a distended gallbladder with diffusely thickened walls, focal ill-defined echogenic mass in the fundus and 13 mm sized calculi in the neck, CBD was 8 mm with smooth distal tapering.

**Figure 1:**
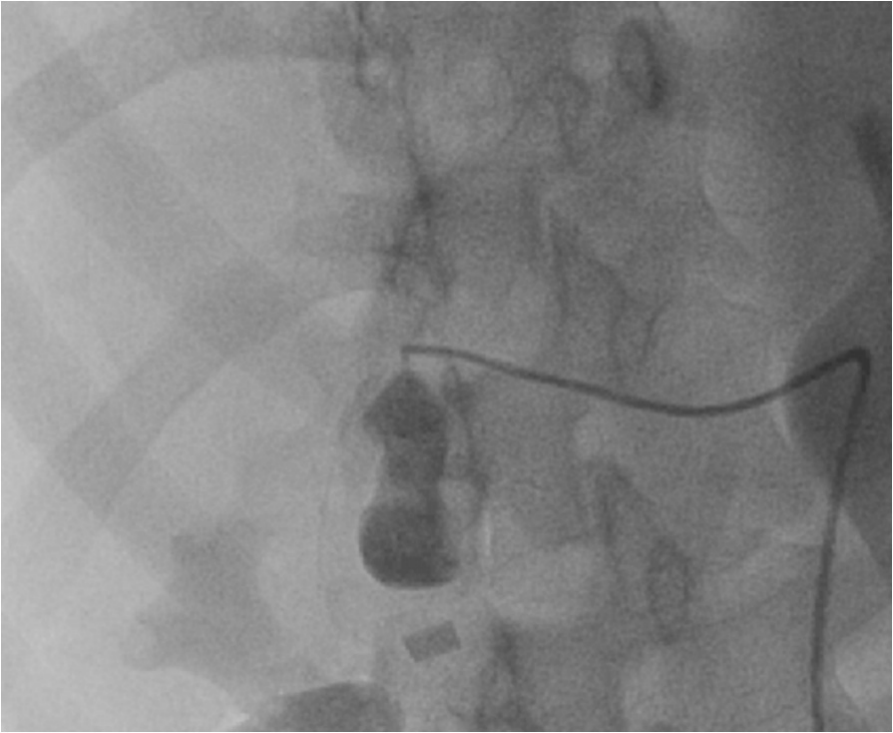
Superselective catheterization of the cystic artery showed extravasation of dye into a sac like structure which was thought to be a pseudoaneurysm.

**Figure 2:**
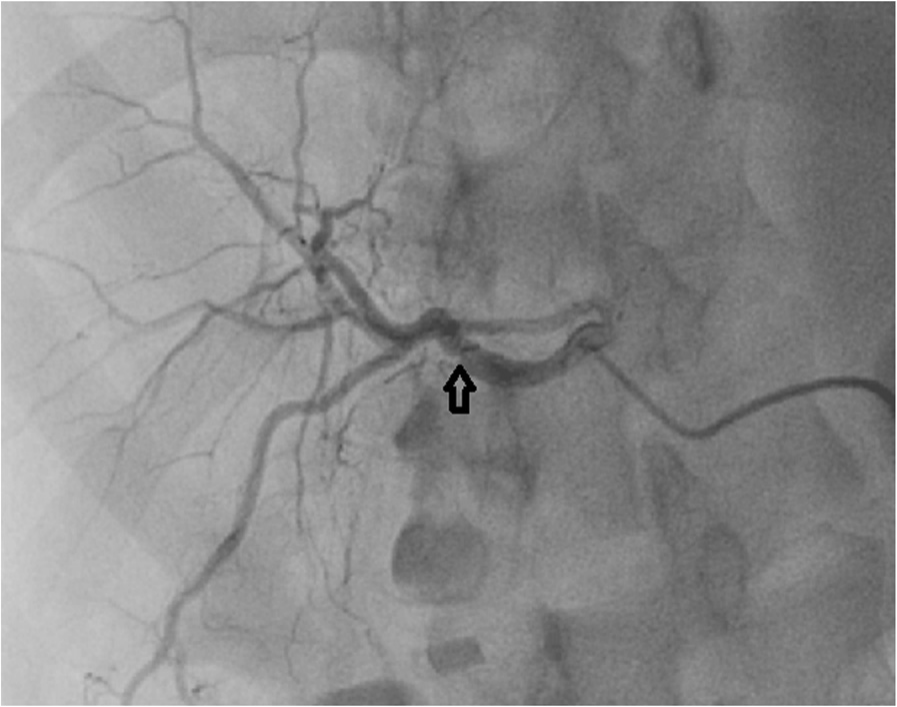
Post coil embolization check angiography showed that the coil had migrated into the right branch of hepatic artery (Arrow).

However subsequent to his admission he developed multiple episodes of vomiting with history of passage of a worm in vomitus, waxing waning jaundice, and episodes of severe haematmesis and melana for which he was further evaluated. His Haemoglobin level decreased to 6 gm/dl, an upper GI endoscopy showed an ulcer with a clot in the first part of duodenum but no active bleed was seen. As the patient continued to have repeated episodes of upper GI bleed an angiography was planned. Additional file 1: Video S1 and Figure 1 shows video of selective hepatic arterial angiography. Selective hepatic artery angiography demonstrated extravasation from the right hepatic artery (RHA) into a sac like structure which was suspected to be a ruptured pseudoaneurysm. Microcoil embolization of feeder artery was tried, however the coil dislodged into the right branch of hepatic artery (Figure 2).

An emergency laparotomy revealed: 1) dense adhesions in Calots triangle 2) dark discoloration of right lobe of liver 3) gallbladder distended with clots 4) short wide cystic duct 5) large stone and blood clots impacted at the cystic duct CBD junction 6) cholecystoduodenal fistula seen corresponding to the site of duodenal ulcer detected in endoscopy 7) mildly dilated CBD, without stones or blood 6) no pseudoaneurysm could be identified. Review of the angiographic video in the context of the operative findings suggested that the sac like structure which was thought to be a pseudoaneurysm was actually infact the gallbladder itself and thus a diagnosis of fistulation of cystic artery into the gallbladder was made. Retrograde cholecystectomy, evacuation of clots, transcystic irrigation of CBD, closure of cholecystoduodenal fistula right hepatic arteriotomy, retrieval of the endo-coil and right hepatic arterial repair was done. The microcoil could be palpated in the right branch of hepatic artery distal to the origin of the cystic artery. Vascular slings were passed in proximal and distal portion of the right hepatic artery. An arteriotomy was performed just proximal to the coil. At the time of arteriotomy no bleeding was observed however immediately after removal of a clot lodged in the proximal segment of the artery profuse bleeding was noticed on loosening of the proximal vascular sling. However still no back bleeding was seen distally, subsequently upon retrieval of the endocoil significant back-bleed was observed. Immediately after repair of the arteriotomy, pulsations returned in the proximal segment of the artery. There was no leak. The elapsed time from embolization to arterial repair was approximately 4 hours.

In the postoperative period the patient developed deep jaundice with significant elevation of bilirubin, SGOT SGPT alkaline phosphatase and amylase, which rapidly improved after 24 hrs and his biochemical parameters normalized within 4 weeks. A follow-up CT angiography performed at 2 weeks was normal demonstrating free flow in the right hepatic artery. Histopathologic examination of the gallbladder showed features suggestive of acute cholecystitis, and an area of granulation tissue was also seen at the site of cholecystoenteric fistula. At six months followup the patient is asymptomatic without any further episode of bleed or jaundice.

## Discussion and conclusion

Non traumatic haemobilia associated with cholecystitis is rare. Acute haemorrhagic cholecystitis [5,6], rupture of arterial pseudoaneurysm [6-9], fistula between cystic/hepatic artery and gallbladder or biliary system, worm infestations of biliary tract [2], vasulitic syndromes [10], anticoagulant drug overdose [11] and lysosomal disorders like metachromatic leukodystrophy [12] have been some of the reported causes.

Arterial pseudoaneurysm a rare cause of haemobilia mostly involves the hepatic or gastroduodenal arteries and less commonly the cystic artery [7-9,13]. Cystic artery pseudoaneurysms usually occur after laparoscopic cholecystectomy or interventions in the biliary tract [3,4]. Spontaneous pseudoaneurysm of the cystic artery is very rare. In an earlier review 13 such cases were identified [14] thereafter we could identify 8 more reports [8,9,13-24]. Such aneurysms have been thought to occur subsequent to inflammatory processes in the vicinity of the vessel and can rupture into the gallbladder cystic duct or bile duct with resultant haemobilia or rarely rupture into the peritoneal cavity [17,25]. Exceptionally they may be incidentally detected in imaging studies [26].

Nontraumatic fistulation of cystic artery into the gallbladder without any discernable pseudoaneurysm has also been reported and is even rarer [27,28] as was also seen in our case. Given the fact that such aneurysms can occur in the oedematous wall of the gallbladder it can be hypothesized that a resultant intramural rupture of such aneurysms can present as a fistula between the cystic artery and gallbladder without an apparent pseudoaneurysm. Worm infestations have been reported to cause haemobilia [2] and an unusual history of passage of worm in the vomitus preceded the acute episode of gastrointestinal bleed in this case. Another cause of non-aneurysmal spontaneous cystic artery bleed reported is due to erosion of a gallbladder ulcer into the cystic artery [29] however no definite ulcer was seen in this case. Sloughing of the gallbladder mucosa as seen in necrotizing infections may also lead to intramural fistulation and haemorrhagic emphysematous cholecystitis [5].

It has been suggested that haemobilia should be one of the differential diagnosis for patients with hepatobiliary disease who present with gastrointestinal bleed [2,3,15]. Our patient initially presented with features of acute cholecystitis but subsequently developed massive gastrointestinal haemorrhage. The distended gall bladder filled with blood can erode into an adjacent bowel viz the duodenum or the colon and result in life threatening gastrointestinal haemorrhage [30-34] or rarely rupture into the peritoneal cavity. A bilioenteric fistula has usually been noted in patients with haemobilia especially in the context of massive gastrointestinal haemorrhage [14,31]. Thus a high index of suspicion of a bilioenteric fistula is warranted in patients with haemobilia and features of massive gastrointestinal haemorrhage.

The clot in the lumen of gall bladder can mimic a mass or a stone. Doppler sonography can aid in the disntinction of a clot from stone or mass by demostrating flow in the gallbladder lumen [7,13-15]. Mere demonstration of blood in gallbladder as in USG does not imply that the bleeding is in the vicinity of the organ as haemobilia from other sites in the hepatobiliary tract can manifest as subsequent haemorrhagic cholecystitis [35]. Endoscopy is the initial investigation of choice in evaluation of upper gastrointestinal haemorrhage. The classic observation of blood coming from inside the papilla has been reported to be diagnostic in haemobilia [7,13,14,19,29]. In this case endoscopy demonstrated an ulcer in the first part of the duodenum with blood clot, similar observations have also been reported in literature where a cholecystoenteric fistula site was confused as a duodenal ulcer [14,32]. Thus finding of a duodenal ulcer in endoscopy often deludes the diagnosis as peptic ulcer aetiology may be sought. However the presence of jaundice in such patients should raise the suspicion.

Selective arterial angiography is helpful in identifying the source of gastrointestinal haemorrhage. The angiographic video seen in our case is an iconic depiction of a fistulous communication between cystic artery and gallbladder lumen. However such a picture being rare it was mistaken for a pseudoaneurysm of the cystic artery, subsequent intraoperative review of the findings clinched the final diagnosis - the gallbladder was filled with blood clots, there was no detectable pseudoaneurysm in the vicinity.

Before the advent of angiographic localization and embolization resectional surgeries of the gastrointestinal tract viz: gastrectomy, cholecystectomy, colectomy and hepatic resections were resorted to in order to control bleeding in haemobilia but were by and large unsuccessful [1]. Surgery has been one of the earliest methods managements of pseudoaneurysms of the cystic or right hepatic artery which have traditionally been treated with arterial ligation [1]. However with the advent of minimally invasive procedures accurate diagnosis and treatment can be rendered by these techniques with less morbidity. Surgery is also used as a salvage option for failed minimally invasive procedures or if there are complications.

The minimally invasive techniques that are available for management of a pseudoaneurysm include: USG guided compression, injection of thrombotic agents into the pseudoaneurysm and or vascular exclusion processes like embolization and stenting [36]. USG guided compression or injection of thrombotic agents is more applicable to extremity rather than visceral aneurysms which are by and large treated with vascular exclusion processes [36]. Angiographic embolization remains the main stay of treatment as also the first step and is considered the gold standard now a days [2]. In early literature embolization of proximal arteries were performed with resultant infarction and organ damage [37]. With the availability of fine catheters super selective catheterization and embolization of the feeder vessel or pseudoaneurysm with micro coils can control the active bleed [37] as also facilitate subsequent laparoscopic cholecystectomy has been recommended [7,8,14,19,29]. Even in open cholecystectomy torrential haemorrhage can occur from accidental rupture of arterial pseudoaneurysm and establishing a proximal control before venturing into the area of pesudoaneurysm is advisable [13]. In an expendable artery with no collateral circulation microcoil embolization of the feeder vessel is recommended like the cystic artery in this case. In arteries with significant collateral circulation if microembolization is attempted both proximal inflow and distal outflow occlusion should be attempted [36]. Vascular stenting can be an alternative to embolization. If the neck of the pseudoaneurysm is wide then stenting should be preferred over embolization. However stenting is difficult in tortuous and small caliber vessels posing a high risk of thrombosis like visceral arteries, besides being costlier [36].

Transcatheter embolization is considered to be a safe procedure with a reported complication of less than 2% [37]. However angiographic embolization can have complications such as necrosis of the gallbladder [14], coil migration with resultant non target embolization, distal dislodgement, thrombosis of parent vessel, perforation and fistulation into viscera/CBD and persistent perfusion through microcoils [37-40]. Microcoil migration is a rare complication of embolization reported to be seen in 0.3% in a series of studied abdominal vascular embolization [40]. There have been various reasons quoted for such a migration after arterial microcoil embolization most important of which include: discrepancy between microcoil size and vessel wall diameter, high blood flow, unstable catheter tip, variation in vessel diameters with respiration the latter particularly in veins [40-42]. The type of microcoil used is also an important factor. Pushable microcoils especially in high flow vessels have a higher chance of incorrect placement and migration as compared to detachable coils and liquid coils [40,41]. The technical challenges that have been reported in microcoil embolization of mesenteric vessels are resultant to atherosclerosis, tortuosity, dual blood supply and small size [37]. Despite the drawbacks a success rate of 80%-90% have been reported with embolization for visceral bleed [37]. Besides metallic microcoil a number of other agents can be used: gelfoam, particulate agents, liquid embolic. Liquid embolics have improved precision permeance and penetration and are thus useful in high flow vessels [41]. Various types of metallic microcoils are available which include: push type, fibred metallic interlocking detachable type with extractable and non extractable variants [41]. The metallic microcoils being radiopaque ease radiographic visualization during angiographic procedures [41].

The options for management of a migrated microcoil include: observation, endovascular extraction [43], parent arterial ligation and operative intervention. Observation may be hazardous in the context of continuing bleed and due to risk of non target embolization. Endovascular extraction with microsnares may be a good option in the newer generation detachable microcoils which are extractable, however the method may not be applicable in older microcoils [41,43]. Ligation of parent artery in which branch arterial ligation has failed has been suggested but there are some limitations: in presence of a collateral flow significant back-bleed can occur, and flow interruption can result in ischaemic damage to the organs.

Animal studies have shown that ligation of the hepatic artery can be fatal, case series of accidental, thrombotic or deliberate occlusion of the hepatic artery in human beings do not corroborate the findings and hepatic necrosis has only rarely been documented. Presence of collateral blood flow, increased portal venous flow, improved oxygen extraction capacity of the liver are some of the mechanisms proposed to protect from long term ischaemic sequel [44,45]. Actually the issue of RHA damage in humans has not been well documented and has only been studied in relation to combined vasculobiliary injuries especially in the context of laparoscopic cholecystectomy. Nevertheless an increased rate of complication has been noted in patients having arterial injury in association with biliary injury [46-48]. Inadvertent ligation/transection of the right hepatic artery though might seem innocuous, can occasionally have hazardous consequences like, slow hepatic infarction, sectoral hepatic necrosis, hepatic abscess and or hepatic duct strictures [46-49]. Damage to the proper hepatic artery or common hepatic artery is reported to cause rapid hepatic infarction [46]. Thus repair of hepatic artery injuries whenever feasible is advised [46-48]. Though hepatic artery ligation remains as one of the option for managing hepatic artery injury or pseudoaneurysms in light of the complications discussed earlier a judicious decision for right hepatic arterial repair is suggested [48,49]. Right hepatic arterial repair can vary from a simple repair, end to end anastomosis and or graft interposition [47,48].

In management of cystic artery pseudoaneurysm most authors resorted to proximal hepatic control and ligation of cystic artery along with early cholecystectomy [8,9,13] however in the face of uncontrolled bleeding a reasonable approach would be to perform a diagnostic angiography and simultaneous intervention for accurate localization of the source of the bleed and immediate control which can be followed by an urgent cholecystectomy [14,19,22].

In our patient super-selective catheterization of the cystic artery and metallic microcoil embolization was attempted, however the microcoil dislodged into the right branch of hepatic artery. Stenting was not attempted in this case a s a first option due to the small caliber of the vessel as also the cost which was prohibitive for our patient. We feel that the discrepancy of the size of microcoil and vessel diameter was the cause of migration in our case due to oversized microcoil. The microcoil that we used was a detachable non retractable type with gel coating thus endovascular extraction was not possible and there was a high chance of thrombosis of the RHA or continued ongoing bleeding therefore a decision for surgical exploration was undertaken for control of haemorrhage as well as for definitive therapy was warranted due to failure to embolize the feeding vessel. Therefore an early exploration was performed. In the absence of a consensus on ligation versus repair of hepatic artery in the situation an arteriotomy for retrieval of the coil along with hepatic artery repair was planned and could be safely accomplished.

Non traumatic pseudoaneurysm or fistula of cystic artery or hepatic artery are rare complications of cholelithiasis and can present with haemobilia complicated by massive gastrointestinal haemorrhage resultant to an associated bilio-enteric fistula. Endoscopically the fistula site may be confused as an ulcer. Angiography is useful in locating the source of the bleed and embolization may be attempted caveat the associated hazards of the procedure. Hepatic artery repair may be a feasible alternative to ligation in such patients but should be judiciously used.

### Consent

Written informed consent was obtained from the patient for publication of this case report and any accompanying images. A copy of the written consent is available for review by the Editor-in-Chief of this journal.

## Abbreviations

Hb: Haemoglobin; TLC: Total leukocyte count; AST: Aspartate aminotransferase; ALT: Alanine amino transferase; Alk PO4: Alkaline phosphatase; CBD: Common Bile Duct; USG: Ultrasonography; CT: Computed Tomography; RHA: Right hepatic artery; GI: Gastrointestinal.

## Competing interests

The authors declare that they have no competing interest.

## Authors’ contributions

All authors have contributed to patient management, writing the case report reviewing and have given the final approval to publishing the manuscript.

## Pre-publication history

The pre-publication history for this paper can be accessed here:

http://www.biomedcentral.com/1471-230X/13/43/prepub

## Supplementary Material

Additional file 1: Video S1 Selective angiography demonstrating extravasation from branch of hepatic artery into a sac like structure confused as a ruptured pseudoaneurysm. Click here for file
